# Immunogenicity of SARS-CoV-2 vaccination in patients undergoing autologous stem cell transplantation. A multicentric experience

**DOI:** 10.3389/fonc.2022.897937

**Published:** 2022-12-02

**Authors:** Francesco Autore, Luca Stirparo, Idanna Innocenti, Elena Papa, Francesco Marchesi, Chiara Togni, Sabrina Mariani, Lorenzo Torrieri, Martina Salvatori, Francesca Fazio, Elisabetta Metafuni, Sabrina Giammarco, Federica Sora, Paolo Falcucci, Antonella Ferrari, Silvia Maria Trisolini, Saveria Capria, Agostino Tafuri, Patrizia Chiusolo, Simona Sica, Luca Laurenti

**Affiliations:** ^1^ Dipartimento di Diagnostica per Immagini Radioterapia Oncologica ed Ematologia Fondazione Policlinico, Universitario A. Gemelli Istituto di Ricovero e Cura a Carattere Scientifico, Rome, Italy; ^2^ Sezione di Ematologia, Dipartimento di Scienze Radiologiche ed Ematologiche, Università Cattolica del Sacro Cuore, Rome, Italy; ^3^ Hematology and Stem Cell Transplant Unit, Regina Elena National Cancer Institute IRCCS, Rome, Italy; ^4^ Hematology Unit, Azienda Ospedaliera Universitaria Sant’Andrea, Sapienza University, Rome, Italy; ^5^ Hematology, Department of Translational and Precision Medicine, Sapienza University, Rome, Italy

**Keywords:** SARSC-CoV-2, vaccination, autologous stem cell transplant (ASCT), efficacy, rituximab

## Abstract

COVID-19 disease has a strong impact on hematological patients; those receiving autologous hematopoietic stem cell transplantation (aHSCT) represent a particularly vulnerable group, in which the effectiveness of vaccination is very variable. Chiarucci et al. showed that patients affected by non-Hodgkin lymphoma (NHL) and treated with rituximab experienced a lower rate of immunization against SARS-CoV-2 (54%), as well as significantly lower IgG antibody titers. In our multicenter retrospective observational study, we included 82 patients who underwent aHSCT, divided into two groups: 58 patients vaccinated after aHSCT (group A) and 24 vaccinated before getting transplantation (group B). In group A, 39 (67%) patients had positive serology, and the rate of positivity increased with time after aHSCT. In the subgroup of patients with NHL, the administration of rituximab predicted negative serology, particularly when administered in the 6 months before vaccination (13% response rate). Patients affected by plasma cells had a higher rate of positivity (83% overall), independently of the time to aHSCT. In group B, no patient who initially showed positive serology became negative after transplantation, so the aHSCT did not affect the response to the vaccination. Our study confirmed the role of rituximab as a negative predictor of response to SARS-CoV-2 vaccination, whereas the conditioning and transplantation procedure itself seemed to be less important.

## Introduction

This study tries to answer questions about how immunotherapy can affect response to SARS-CoV-2 vaccination and to define the role of conditioning, the kind of hematological disease, and the timing of SARS-CoV-2 vaccination.

Since COVID-19 disease was declared a pandemic by the World Health Organization (WHO) in March 2020, patients with hematological diseases and even more patients undergoing autologous HSCT were strictly observed as they were considered high-risk patients.

In a retrospective study of 134 patients undergoing aHSCT, overall survival (OS) at 30 days from the diagnosis of COVID-19 was 67% (1). These data showed the need for effective primary prevention, such as vaccination, in fragile patients. However, the effectiveness of vaccination in self-transplanted patients seems to be very variable but overall lower than that in the general population (2). Most vaccinations are recommended at least 3–6 months after aHSCT and 6–12 months after an anti-B-cell antibody therapy (such as rituximab, RTX), given the strong inhibition of the response to vaccination that results after the administration of those therapies (3). Since December 2020, several vaccine platforms against SARS-CoV-2 have been developed with a high efficacy and safety profile (4, 5). Currently, the Italian Society of Hematology (SIE) and Italian Bone Marrow Transplant Group (GITMO), recommend vaccination for COVID-19 in aHSCT patients starting from 3 months post-transplant, with the preferential use of an mRNA vaccine (6).

As of today, few studies have been carried out regarding the response to SARS-CoV-2 vaccination in patients undergoing aHSCT.

The Spanish Hematopoietic Stem Cell Transplantation and Cell Therapy Group performed an analysis on 84 patients who underwent aHSCT (16 of whom were affected by NHL): variables associated with lower rates of detection in aHSCT recipients were active treatment with corticosteroids and NHL as underlying disease (60% of positivity rate *vs* 91% in patients with multiple myeloma MM) (7).

Maneikis et al. performed a national prospective cohort study in patients with hematological malignancies in Lithuania (8); patients who underwent either allogeneic HSCT or autologous HSCT (122 and 192 patients, respectively) had low serological responses within the first 6 months of HSCT but improved afterwards. Responses in the group of patients who received RTX were low within the first 12 months since last treatment; beyond 12 months after last treatment, serological responses improved but remained heterogeneous.

A recent article published by Chiarucci et al. (9) compared serological data from 50 HSCT recipients (38 autologous and 12 allogeneic) and 45 healthy subjects, collected 30 days after their vaccination against SARS-CoV-2. In patients receiving high-dose therapy and aHSCT, prior administration of RTX was associated with a lower rate of immunization against SARS-CoV-2 (54%), as well as a significantly lower IgG antibody titer. Notably, only 20% of patients receiving the mRNA-based SARS-CoV-2 vaccine BNT162b2 within a year of the last dose of RTX developed a protective antibody titer.

Therefore, we collected data from patients who underwent aHSCT in four centers to confirm these results and find out other important features regarding vaccination in the setting of aHSCT. Based on these data, we suppose that the time between aHSCT and vaccination or other previous therapies affects the immunogenicity. We also want to confirm the negative role of RTX in this context and clarify how the timing of the last administration could affect the response to vaccination. PECO formulation of objectives: how does the time from aHSCT or last RTX with respect to vaccination against SARS-CoV-2 affect the production of IgG against the spike protein in a population of people affected by hematological malignancies such as lymphomas or myelomas?

## Methods

**Figure 1:**
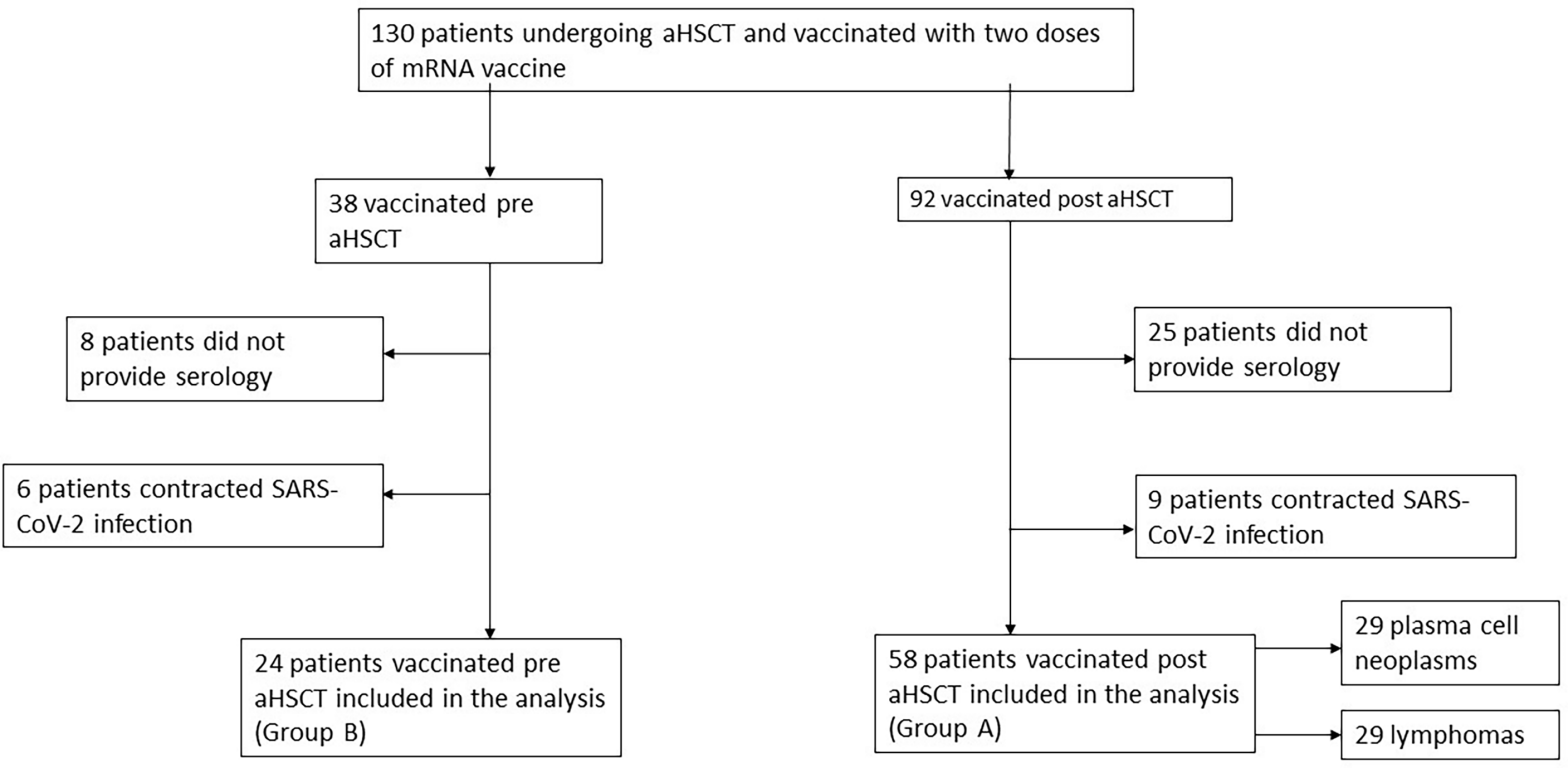
Flow diagram regarding the description of inclusion and exclusion criteria of the study.

This multicenter retrospective observational study included 82 patients who underwent aHSCT from January 2020 to October 2021. Patients were recruited during this period. Exclusion criteria were prior SARS-CoV-2 infection, absence of a second dose of vaccination, and lack of available serology. Patients vaccinated after the aHSCT were defined as group A patients, whereas patients vaccinated before the aHSCT were defined as group B patients. Then, group A patients were divided in two subgroups according to the hematological disease. A flow diagram regarding the description of these patients is represented in **Figure 1**. All four centers participating in this study are in Rome, Italy, and provided data on patients derived from local clinical reports, selected by the already reported inclusion and exclusion criteria. All the data were collected in a database. The post-vaccination antibody tests were variable in different centers, and the measurement methods were different. We considered positive the data in AU/ml or BAU/ml, if provided, if it was above the laboratory range of positivity. The quantitative assays used in this study were provided by ABBOTT SARS-CoV-2 IgG II Quant, using chemiluminescence microparticle immunoassay (CMIA, 15 patients), LIAISON^®^ SARS-CoV-2 S1/S2 IgG by DiaSorin^®^, using chemiluminescent immunoassay (CLIA, 14 patients), and Roche Diagnostics Elecsys^®^ Anti-SARS-CoV-2, using electrochemiluminescence immunoassay (ECLIA, 3 patients). The cutoffs for positivity for each method were, respectively, 50 AU/ml for ABBOTT, 15 AU/ml for DiaSorin^®^, and 0.8 AU/ml for Roche Diagnostics. We used a conversion factor between arbitrary units (AU) of different assays and the WHO international binding antibody unit (BAU) to compare the level of antibodies. Based on the manufacturer’s technical manual, the conversion factors were 0.142 for the ABBOTT assay, 2.6 for the DiaSorin^®^ assay, and 0.97 for the Roche^®^ Diagnostics assay. A total of 15 patients were evaluated only with a qualitative immunoassay, mainly using ELISA and ECLIA, and 11 patients provided serology from an external laboratory without information on the method. Moreover, quantitative data were not collected for patients vaccinated before transplantation. All patients included in this registry signed an informed consent according to the Declaration of Helsinki. Demographic and clinic characteristics are summarized in **Table 1**. All patients were vaccinated against SARS-CoV-2 with two doses of 30 mcg of a mRNA vaccine (BNT162b2 or mRNA-1273). In both groups, we considered only the serologies provided after the second dose of vaccine, conducted at least three weeks after vaccination.

**Table 1:** Demographic, clinical characteristics, and seropositivity rates of patients who underwent aHSCT.

	Patients vaccinated after aHSCT (%)Group A	Seropositivity rates (%)Group A	Patients vaccinated before aHSCT (%)Group B
**All patients**	58	39 (67)	24
**Sex**			
Male	29 (50)	17 (59)	14 (58)
Female	29 (50)	21 (72)	10 (42)
**Age**			
median	59 (27-71)		59 (28-69)
≤ 60	30 (52)	18 (62)	15 (62)
>60	28 (48)	20 (69)	9 (38)
**Diagnosis**			
Non-Hodgkin lymphomas	26 (45)	13 (50)	7 (29)
Hodgkin lymphomas	3 (5)	2 (67)	4 (17)
Multiple myeloma	28 (48)	24 (86)	13 (54)
Plasma cell leukemia	1 (2)	0 (0)	
**Time between aHSCT and vaccination**			
Median (days)	240 (18–510)		57 (5–210)
≤6 months	21 (37)	12 (57)	23 (96)
>6 months ≤12	28 (48)	19 (68)	1 (4)
>12 months	9 (15)	8 (89)	
**Time between vaccination and serology**			
Median (days)	65 (24-214)		124 (22–234)
**Time between aHSCt and serology (only for group B)**			
Median			60 (15–165)
**Prior rituximab administration (NHL only)**			
Yes	24 (92)		5 (71)
No	2 (8)		2 (29)
**Time between rituximab and vaccination**			
≤6 months	8 (33.3)	1 (13)	5 (100)
>6 months ≤12	8 (33.3)	4 (50)	
>12 months	8 (33)	7 (87)	

aHSCT, autologous hematopoietic stem cell transplantation; NHL, non-Hodgkin lymphoma.

### Statistical analysis

Patients’ characteristics were summarized using cross-tabulations for categorical variables or using quantiles for continuous variables. Data distribution was tested by distribution tests (normal, lognormal, and gamma). Logistic regression models were used in univariate and multivariate analyses to assess the effect of factors on the seropositivity dichotomous variable. Odds Ratios (ORs) and 95% Confidence Intervals were reported as parametric results of the logistic regression models in univariate and multivariate analyses. Gamma models were used to assess the effect of factors on the quantitative values of seropositivity. All tests were two-sided, accepting p <0.05 for a statistically significant difference. All analyses were performed using R software (R Core Team, 2021. R: A language and environment for statistical computing. R Foundation for Statistical Computing, Vienna, Austria).

## Results

Of the 82 patients enrolled in the study, 58 patients were vaccinated after autologous transplantation (group A) and 24 patients before (group B). Patients vaccinated after aHSCT provided a single serology test showing an antibody response against SARS-CoV-2 spike glycoprotein at least one month after vaccination with the second dose. We divided patients in group A into two subgroups according to the hematological disease: 29 patients affected by plasma cell neoplasms (28 multiple myeloma and 1 plasma cell leukemia) and 29 patients affected by lymphomas (26 non-Hodgkin lymphoma, NHL, and 3 Hodgkin lymphoma, HL). We then compared patients according to their conditioning regimen and previous treatment.

**Figure 2:**
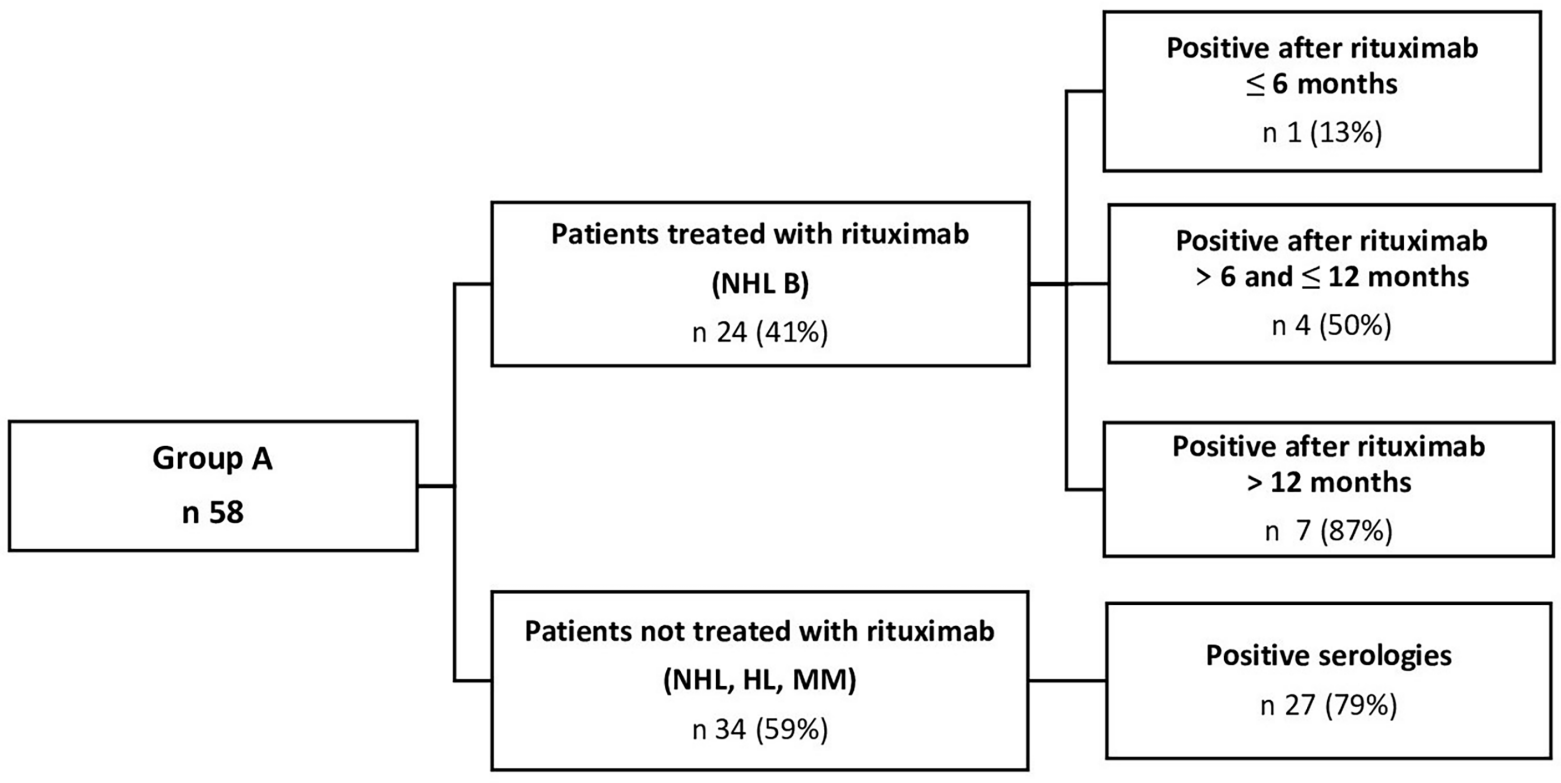
Comparison of positive serologies between patients treated and not treated with Rituximab when vaccinated after aHSCT (group A).

In group A, including 58 patients vaccinated after transplantation (**Figure 2**), 39 (67%) had positive serology, and 19 (33%) did not develop an antibody response. We noticed that the rate of positivity increased with time after aHSCT. When vaccination was performed in the first 6 months after the transplantation, only 57% of patients tested positive, but this value increased to 68% when considering patients vaccinated after 6 and within 12 months, and to 89% after 12 months. The subgroup of patients affected by lymphomas had a lower rate of positivity (52%), even if two out of three cases (67%) of HL were positive. In the group of lymphomas, the distance from the aHSCT was particularly significant: in fact, the positivity was 30% when the vaccination was performed within 6 months, 54% after 6 and within 12 months, and 83% after 12 months. When considering NHL, the distance from the administration of RTX had an impact on the vaccination response: patients receiving the monoclonal antibody within 6 months had a poorer response to vaccination (13% of positivity) than those who were treated with RTX after 6 and within 12 months (50%), and after 12 months (87%). In the plasma cell neoplasms subgroup, the rate of positivity was higher (83% overall), and it was not particularly affected by the time from transplant; in fact, results showed a rate of 82% even in patients vaccinated within 6 months. When considering the conditioning regimen, it’s worth noting that the ones used for the lymphomas had a lower positivity rate: 52% for FEAM (10) (Fotemustine, Etoposide, Cytarabine, and Melphalan); 33% for BEAM (Carmustine, Etoposide, Cytarabine, and Melphalan); and 67% for BuMel regimen (Busulfan plus Melphalan) than those used for the myelomas (100% for Melphalan 140 mg/m^2^ and 81% for Melphalan 200 mg/m^2^ (11)). These differences between conditioning regimens may be explained by the highest prevalence in the use of FEAM for lymphomas (23 out of 29 patients) and Mel 200 for myelomas (27 out of 29).

The logistic regression model (**Supplementary Table 1**) showed an increased rate of seropositivity in patients with plasma cell disorders (OR 4.48, CI 1.41–16.3, p-value of 0.015), and the conditioning regimen showed a lower positivity rate in patients who did not undergo the Mel regimen (OR 0.22, CI 0.06–0.71, p-value of 0.015). Administration of RTX also had a negative impact on immunogenicity (OR 0.26, CI 0.08–0.8, p-value of 0.022). When considering time, we found out that the distance between aHSCT and vaccine did not increase seropositivity (OR 1.18, CI 1.02–1.41, p-value of 0.04), particularly after 12 months, even if this partition of time (≤6 months, between 7 and 12 months, and > 12 months) did not reach statistical significance for any disease (OR 6, CI 0.86–122, p-value of 0.12). The multivariate analysis confirmed a correlation between seropositivity and time (OR 1.21, CI 1.03–1.46, p-value of 0.028) or pathology (OR for plasma cell neoplasms 5.34, CI 1.56–21.3, p-value of 0.011).

We then analyzed the number of antibodies developed after vaccination in the 32 patients who had quantitative serology converted into BAU/ml. The mean level of antibodies was 139.533 BAU/ml overall, with a wide range (from 0.02 to 11,097). Considering each assay, the mean value for ABBOTT was 80.72 BAU/ml (0.02 to 11,097), for the DiaSorin^®^ assay, it was 277.07 BAU/ml (9.88 to 10,504), and for the Roche diagnostic assay, it was 284.07 BAU/ml (11.49 to 1,421). When considering the time from transplantation to vaccination, the mean value of antibody levels was 68.05 BAU/ml (0.143 to 7,046) when the vaccine was done within 6 months from transplantation, 139.82 BAU/ml (0.02 to 7,310) between 7 and 12 months, and 1,035.97 BAU/ml (11.49 to 11,097.94) after 12 months (**Supplementary Figure 1**). We also analyzed these data in the RTX group (**Supplementary Figure 2**), with a mean value of 96.74 BAU/ml (0.143 to 11,097). When vaccination was administered within 1 year of the last RTX, the mean value of antibodies was 6.78 BAU/ml (0.143 to 1,421), whereas after a year from the last administration it increased to 1,379.42 BAU/ml (11.49 to 11,097). When performing statistical analysis on quantitative data, a gamma distribution was considered, and the test was passed (p-value = 0.7795). No statistically significant associations were found in the univariate generalized linear models (**Supplementary Table 2**).

**Figure 3:**
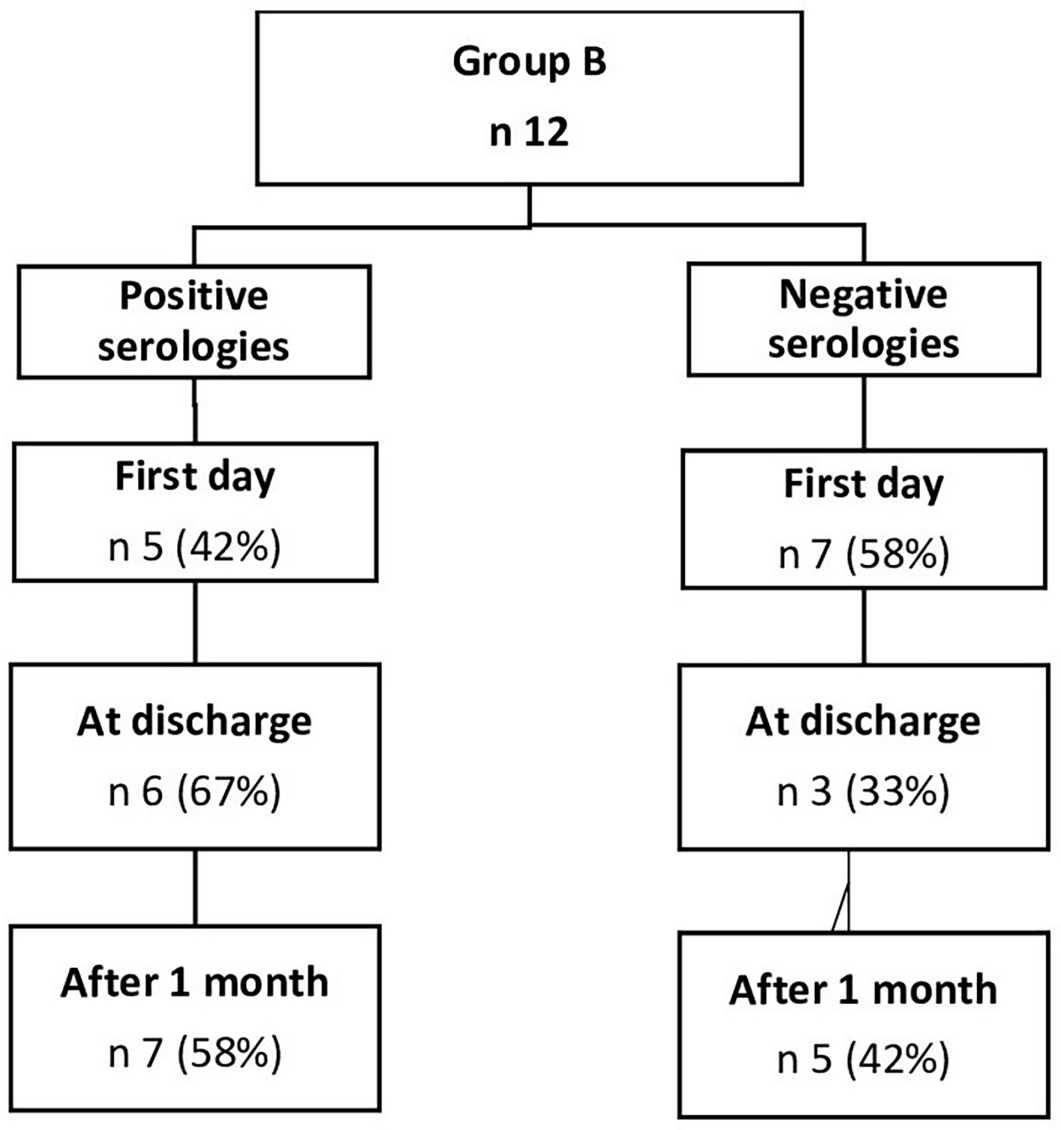
Serologies from patients vaccinated before aHSCT (group B).

In group B, 12 patients were tested on the first day of hospitalization, at discharge, and 1 month after transplantation, while the other 12 patients provided just the serology 1 month after the aHSCT. In this group, 15 patients (63%) were positive at least 1 month after the aHSCT, while nine patients (37%) were negative. We also noticed that, in the subgroup of patients tested both at hospitalization and discharge, no patient who was initially positive became negative after transplantation (**Figure 3**). Patients affected by NHL had a lower rate of positivity (45%) compared to those affected by plasma cell neoplasms, who showed 77% positivity.

## Discussion

We confirmed the role of RTX as a negative predictor of response to SARS-CoV-2 vaccination in patients affected by lymphoproliferative disorders and plasma cell neoplasms who underwent aHSCT, as already reported by Chiarucci et al. (9) and Maneikis et al. (8). Notably, only one patient out of eight vaccinated in our series within 6 months of the last RTX showed positive serology (13%). On the other hand, patients without RTX administration (NHL, HL, and plasma cell neoplasms) had a vaccination response in 71% of cases.

Therefore, our cohort of patients affected by NHL had a poor response, according to what was reported by Piñana et al. (7) (50% and 60% overall responses, respectively), probably due to the treatment with anti-CD20 monoclonal antibodies, even if they did not analyze this specific aspect.

We also confirmed the better response of patients affected by plasma cell neoplasms, who responded in 83% of cases in our cohort, similarly to those of previously cited studies, even if slightly inferior [95% in Chiarucci et al. (9), 91.8% in Piñana et al. (7)].

When considering the analysis of serological values, our data did not show a real progression of values in time between 6 months and 1 year after transplantation, whereas the values of antibodies were higher after 1 year. The mean level of antibodies in patients treated with RTX was confirmed to be lower than the mean overall value (96.74 BAU/ml *vs* 139.533 BAU/ml).

The comparison between serologies from patients vaccinated within and after 1 year from the last RTX was much more like what we observed in the qualitative analysis, with an important progression of values after 1 year from the last RTX.

In conclusion, we confirmed the crucial role of RTX and the scant importance of a conditioning regimen in the response to vaccination in patients affected by lymphoproliferative diseases who undergo aHSCT. According to these data, it would be more useful to consider vaccination at least 6 months after the last RTX administration. However, it is necessary to consider the patient’s condition and the epidemiological context.

Therefore, our study shows a limited role for aHSCT in determining the serologic response to vaccination in this setting of patients.

Limitations of this study may include the small size of samples in each group and the fact that we could not collect all the quantitative antibody titers. In contrast, we focused attention on previous therapies, especially monoclonal antibodies targeting CD20 such as RTX, rather than transplantation conditioning itself. Moreover, we added specific timelines between the aHSCT and the vaccination and their relative responses, to better understand when the vaccination could be more effective.

## Data availability statement

The raw data supporting the conclusions of this article will be made available by the authors, without undue reservation.

## Ethics statement

The studies involving human participants were reviewed and approved by the Fondazione Policlinico Gemelli. Prot N 0040327/21. The patients/participants provided their written informed consent to participate in this study.

## Author contributions

FA, LL, and LS designed the study and wrote the manuscript. EP, FM, CT, SM, LT, MS, FF, EM, SG, FS, and SMT collected the data. LS performed statistical analysis. II, PF, AF, SC, AT, PC, SS, and LL supervised the study and approved the final version of the manuscript. All authors contributed to the article and approved the submitted version.

## Conflict of interest

The authors declare that the research was conducted in the absence of any commercial or financial relationships that could be construed as a potential conflict of interest.

## Publisher’s note

All claims expressed in this article are solely those of the authors and do not necessarily represent those of their affiliated organizations, or those of the publisher, the editors and the reviewers. Any product that may be evaluated in this article, or claim that may be made by its manufacturer, is not guaranteed or endorsed by the publisher.
