# Supplementary figures and images for: Immunogenicity of SARS-CoV-2 vaccination in patients undergoing autologous stem cell transplantation. A multicentric experience
Source: Front Oncol. 2022 Dec 2;12:897937. doi: 10.3389/fonc.2022.897937 (PMC9755510; doi:10.3389/fonc.2022.897937)

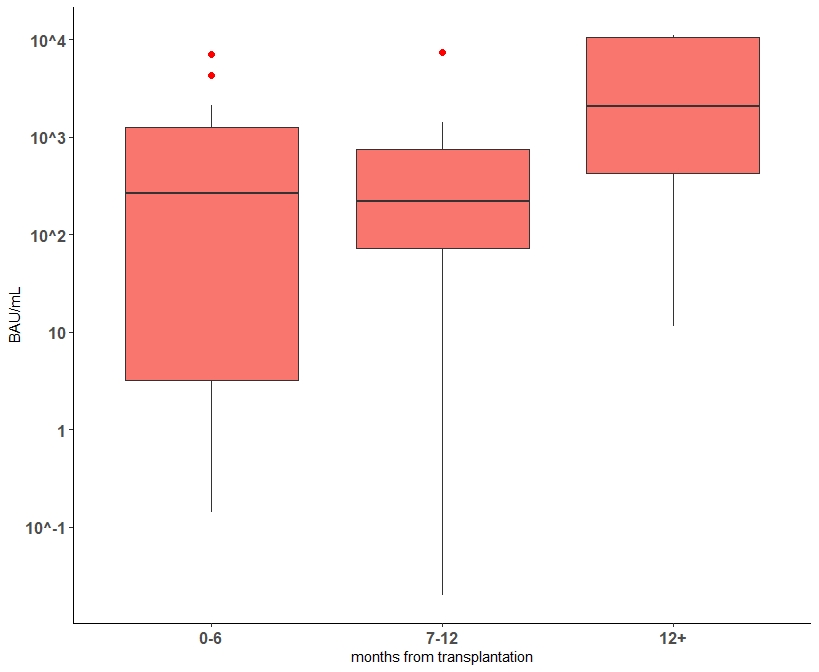

Supplement: Supplementary file 1 [file Image_1.jpeg]

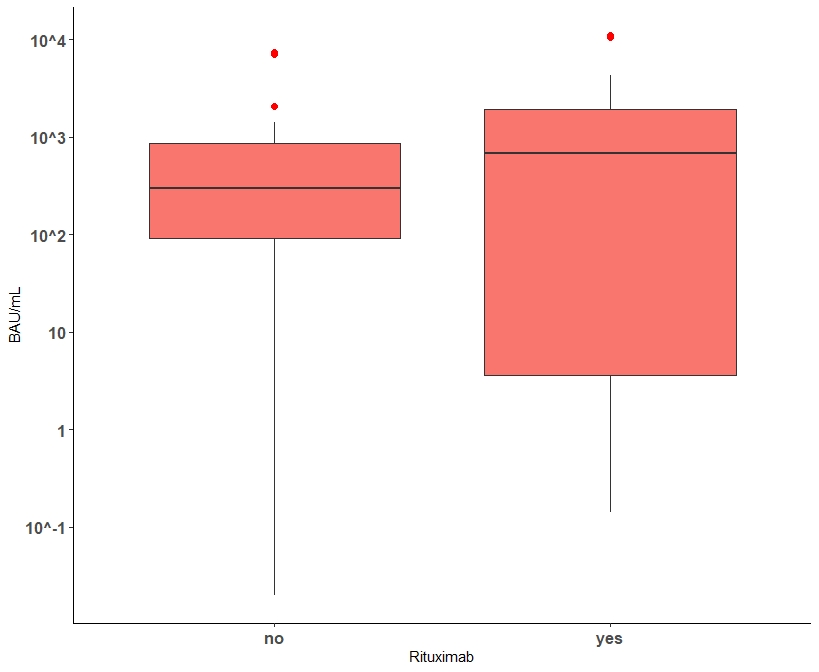

Supplement: Supplementary file 2 [file Image_2.jpeg]
