# Supplementary material for: Immunogenicity of SARS-CoV-2 vaccination in patients undergoing autologous stem cell transplantation. A multicentric experience
Source: Front Oncol. 2022 Dec 2;12:897937. doi: 10.3389/fonc.2022.897937 (PMC9755510; doi:10.3389/fonc.2022.897937)
Supplement: Supplementary file 3 [file Table_1.docx]

|  | Univariate | | | | Multivariate | | |
| --- | --- | --- | --- | --- | --- | --- | --- |
| Characteristic | N | OR^1^ | 95% CI^1^ | p-value | OR^1^ | 95% CI^1^ | p-value |
| **Pathology** | 58 |  |  |  |  |  |  |
| Lymphoma |  | — | — |  | — | — |  |
| Plasma cells neoplasm |  | 4.48 | 1.41, 16.3 | **0.015** | 5.34 | 1.56, 21.3 | **0.011** |
| **Rituximab administration (yes or no)** | 58 | 0.26 | 0.08, 0.80 | **0.022** |  |  |  |
| **Conditioning** | 58 |  |  |  |  |  |  |
| MEL |  | — | — |  | — | — |  |
| no MEL |  | 0.22 | 0.06, 0.71 | **0.015** |  |  |  |
| **Time between aHSCT** | 58 | 1.18 | 1.02, 1.41 | **0.040** | 1.21 | 1.03, 1.46 | **0.028** |
| **Time partition** | 58 |  |  |  |  |  |  |
| 0-6 months |  | — | — |  | — | — |  |
| 7-12 months |  | 1.58 | 0.49, 5.21 | 0.44 |  |  |  |
| 12+ months |  | 6.00 | 0.86, 122 | 0.12 |  |  |  |
| ^1^OR = Odds Ratio, CI = Confidence Interval | | | | | | | |
