# Supplementary material for: Immunogenicity of SARS-CoV-2 vaccination in patients undergoing autologous stem cell transplantation. A multicentric experience
Source: Front Oncol. 2022 Dec 2;12:897937. doi: 10.3389/fonc.2022.897937 (PMC9755510; doi:10.3389/fonc.2022.897937)
Supplement: Supplementary file 4 [file Table_2.docx]

| Characteristic | N | Beta | 95% CI^1^ | p-value |
| --- | --- | --- | --- | --- |
| **Pathology** | 32 |  |  |  |
| lymphoma |  | — | — |  |
| Plasma cells neoplasm |  | 0.0007 | -0.0001, 0.0020 | 0.18 |
| **Rituximab administration (yes or no)** | 32 | -0.0004 | -0.0013, 0.0004 | 0.34 |
| **Conditioning** | 32 |  |  |  |
| MEL |  | — | — |  |
| no MEL |  | -0.0007 | -0.0020, 0.0001 | 0.18 |
| **Time between aHSCT** | 32 | -0.0001 | -0.0001, 0.0000 | 0.065 |
| **Time partition** | 32 |  |  |  |
| 0-6 months |  | — | — |  |
| 7-12 months |  | 0.0002 | -0.0011, 0.0015 | 0.74 |
| 12+ months |  | -0.0006 | -0.0015, 0.0002 | 0.15 |
| ^1^CI = Confidence Interval | | | | |
